# Apex Resection in Zebrafish (*Danio rerio*) as a Model of Heart Regeneration: A Video-Assisted Guide

**DOI:** 10.3390/ijms22115865

**Published:** 2021-05-30

**Authors:** Ditte Gry Ellman, Ibrahim Mohamad Slaiman, Sabrina Bech Mathiesen, Kristian Skriver Andersen, Wolfgang Hofmeister, Elke Annette Ober, Ditte Caroline Andersen

**Affiliations:** 1 DCA-Lab, Department of Clinical Biochemistry and Pharmacology, Odense University Hospital, J. B. Winsløwsvej 25, 1. Floor, 5000 Odense C, Denmark; dellman@health.sdu.dk (D.G.E.); slaiman.ibrahim@gmail.com (I.M.S.); sbmathiesen@health.sdu.dk (S.B.M.); ksandersen@health.sdu.dk (K.S.A.); wolfganghofmeister1@gmail.com (W.H.); 2 DCA-Lab, Institute of Clinical Research, University of Southern Denmark, J. B. Winsløwsvej 19, 5000 Odense C, Denmark; 3 Faculty of Health and Medical Sciences, DanStem (Novo Nordisk Foundation Center for Stem Cell Biology), Blegdamsvej 3B, 2200 København H, Denmark; elke.ober@sund.ku.dk

**Keywords:** regeneration, heart, zebrafish, apex resection, surgical videos, anaesthesia

## Abstract

Ischemic heart disease is one of the leading causes of deaths worldwide. A major hindrance to resolving this challenge lies in the mammalian hearts inability to regenerate after injury. In contrast, zebrafish retain a regenerative capacity of the heart throughout their lifetimes. Apex resection (AR) is a popular zebrafish model for studying heart regeneration, and entails resecting 10–20% of the heart in the apex region, whereafter the regeneration process is monitored until the heart is fully regenerated within 60 days. Despite this popularity, video tutorials describing this technique in detail are lacking. In this paper we visualize and describe the entire AR procedure including anaesthesia, surgery, and recovery. In addition, we show that the concentration and duration of anaesthesia are important parameters to consider, to balance sufficient levels of sedation and minimizing mortality. Moreover, we provide examples of how zebrafish heart regeneration can be assessed both in 2D (immunohistochemistry of heart sections) and 3D (analyses of whole, tissue cleared hearts using multiphoton imaging). In summary, this paper aims to aid beginners in establishing and conducting the AR model in their laboratory, but also to spur further interest in improving the model and its evaluation.

## 1. Introduction

In response to injury such as myocardial infarction (MI), the mammalian heart may compensate by changing its form and function, however the damaged myocardium exhibits limited regeneration with respect to the replenishment of dead cardiomyocytes [1]. Instead, the lost cardiomyocytes are replaced by scar tissue, which may lead to aberrant ventricular remodelling, disrupting proper pump function and ultimately leading to heart failure [1,2], a major challenge globally. The inability to restore the pool of cardiomyocytes and integrate them into the myocardium is considered a major obstacle in rebuilding cardiac function [1].

In contrast to mammals, the adult zebrafish heart has a marked capacity for self-restoration after injury [3,4]. This was first demonstrated in 2002 by Poss et al., who showed that the heart gained full contractile function and appeared fully regenerated 60 days after resecting 10–20% of the ventricular apex [3]. This model is referred to as the apex resection (AR) model. Other models of cardiac regeneration in zebrafish include cryoinjury [5] and genetic ablation [6]. The use of zebrafish in general as disease models offers many advantages and its use is increasing. Compared to other laboratory animals, zebrafish are relatively easy to handle, breed in high numbers, are inexpensive in maintenance and simple to genetically manipulate. Even though the zebrafish seems very distant from humans, they exhibit a similar genetic structure, sharing approximately 70% of annotated genes [7]. Indeed 82% of all genes known to be associated with human disease have an equivalent gene in the zebrafish [7], supporting zebrafish as a valid model for human disease.

The process of heart regeneration after AR may be divided into different steps. Initially after AR a fibrin clot is formed, whereafter a cascade of immune responses and transient scarring occurs. The peri-/epicardium also starts remodelling [8,9,10], and then gradually new myocardium is grown through proliferation of existing cardiomyocytes [8,11,12]. This process is supported by epi- and endo-cardial cells [8,9] and also involves the vascularization and electrical coupling of the cardiomyocytes [3,4,8,9,11,12,13]. In mammals, only low levels of cardiomyocyte proliferation have been observed after injury [14,15,16], while growing evidence underscores the importance of epicardial cells in stimulating cardiomyocyte proliferation in both mammals and zebrafish [17,18,19].

Thus, AR in zebrafish is a valuable model for studying cardiomyocyte proliferation and heart regeneration in order to understand and potentially unlock the regenerative response in mammals. The huge interest in the AR model is reflected in our recent systematic overview of AR zebrafish studies where many different research groups globally have improved the understanding of heart regeneration through cardiomyocyte proliferation [20]. However, during the systematic data retrieval we noticed several variations in the AR procedures and designs that complicate interstudy comparison. For instance, the inclusion of an AR sham procedure is poorly described in the published literature, which may impact conclusions, especially since the tearing peri-/epicardium by itself seems to have a rather high effect on heart regeneration [8,10]. Recently, Wang and Poss [6] and Kikuchi et al. [21], two pioneers within this field of research, provided very informative papers on how to perform AR. However, affecting especially the unexperienced zebrafish researcher, no videos showing the procedures have been published and details regarding anaesthesia also seem to be lacking. Indeed, zebrafish are very sensitive towards anaesthetics, and the anaesthetic procedure thus needs careful attention. Previously, different types of anaesthesia have been tested in zebrafish, including ethyl 3-aminobenzoate methanesulfonate (Tricaine) which is the most commonly used for anaesthetizing zebrafish [22]. Many studies use 0.02% Tricaine to achieve stage 3 anaesthesia [6,21], while others use higher concentrations [5], but information is limited on this important part of the model.

Here we provide a complete protocol for AR in zebrafish, including video footage showing the entire resection procedure and a sham operation. Furthermore, we elaborate on crucial steps such as anaesthesia that increase zebrafish survival and the control design required for interstudy reproducibility. We also suggest and test a new approach to performing subsequent analysis of heart outgrowth taking the whole 3D structure of the heart into consideration. We thus hope this article will ease entry into using the AR in zebrafish as a model for studying heart regeneration.

## 2. Results

### 2.1. Finetuning Anaesthesia in Zebrafish

**Figure 1:**
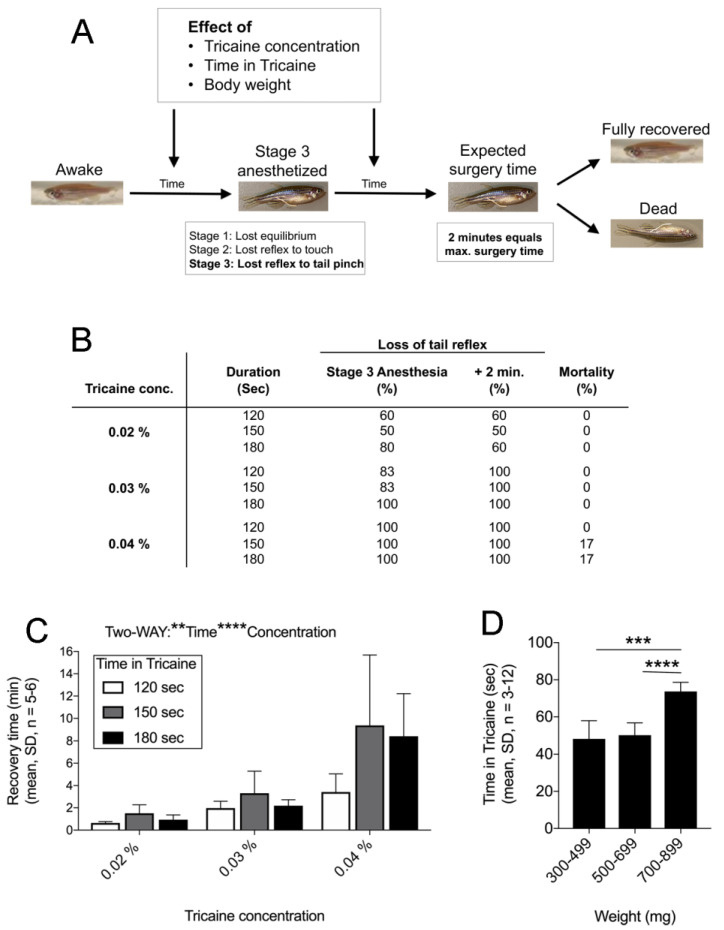
Anaesthesia depends on time and bodyweight of zebrafish. (**A**) Schematic representation of experimental design. Recovery was tested after the indicated time in Tricaine plus 2 min expected surgery time. (**B**) Testing the effect of Tricaine concentrations (anaesthesia, rows) and durations (second column) on time to loss of tail reflex—stage 3 (third column) and after (fourth column) +2 min surgery simulation. The zebrafish mortality was noted for each group. (**C**) Time required to recover after 120 (white bar), 150-(grey bar) or 180 s (black bar) in 0.02, 0.03 or 0.04% Tricaine is depicted, respectively. (**D**) Time needed to reach stage 3 anaesthesia was measured for each zebrafish weight-class (300–499 mg, 500–699 mg or 700–899 mg). Statistical analysis used include (**C**) two-way ANOVA and (**D**) one-way ANOVA with Tukey’s multiple post-test (** *p* ≤ 0.01, *** *p* ≤ 0.001, **** *p* ≤ 0.0001).

Anaesthesia is not only crucial during surgery, but it also has a major effect on zebrafish revival and survival and thus data outcome [23]. During anaesthesia different stages are defined [24] (Figure 1A, lower left box). Stage 1 describes the loss of equilibrium (rightening reflex) with more than 3 s in dorsal recumbency (belly up). Stage 2 concerns the absence of a reflex to a gentle touch while stage 3 defines the loss of reflex to tail pinching with forceps [24], at which stage the zebrafish is ready for surgery (Figure 1A). Tricaine is currently one of the best options regarding fast recovery time and reproducibility compared to other agents [24,25]. Although several studies use 0.02% Tricaine to achieve stage 3 anaesthesia [6,21], we observed that not all zebrafish entered stage 3 anaesthesia for zebrafish with 0.02% Tricaine. Hence, we set out to test Tricaine concentrations (0.02%, 0.03% and 0.04%, Figure 1B) and duration (120, 150 and 180 s, Figure 1B) to define the optimal anaesthesia protocol in regard to establishing and sustaining stage 3 anaesthesia, survival rate, and recovery time. Stage 3 anaesthesia was achieved and maintained for 120 s in 50–80%, 83–100% and 100% of the zebrafish when using 0.02%, 0.03% or 0.04% Tricaine, respectively (Figure 1B). After 120 s in the surgery set up 50–60% of the zebrafish in 0.02% Tricaine, but 100% of zebrafish in 0.03% and 0.04% Tricaine sustained loss of tail reflex (Figure 1B). There was no difference in recovery time between duration in Tricaine at 0.02% and 0.03% (Figure 1C) and no mortality was observed (Figure 1B). However, when using 0.04% Tricaine recovery time (Figure 1C) and mortality increased (Figure 1B). We also investigated how the weight of the zebrafish affected the time required to achieve stage 3 anaesthesia (Figure 1D). There was no difference in time needed in Tricaine between zebrafish of 300–499 mg and 500–699 mg. However, zebrafish above 699 mg required significantly longer time in Tricaine to reach stage 3 anaesthesia.

Thus, based on zebrafish survival and the ability to maintain stage 3 anaesthesia (See materials and methods, and Supplementary Video S1) we proceeded with 0.03% Tricaine and selected for zebrafish with a weight between 300–700 mg to avoid premature awakening.

### 2.2. Apex Resection (AR) in Zebrafish Step-by-Step

**Figure 2:**
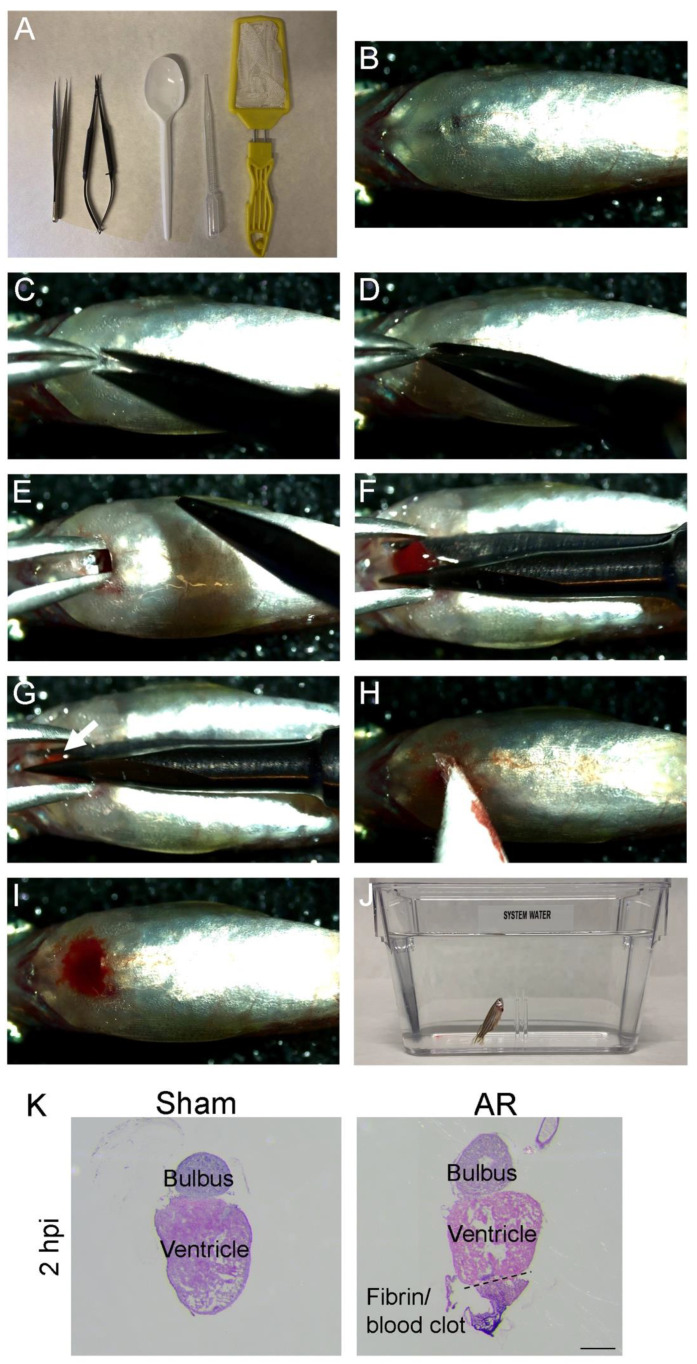
Surgical procedure for sham operation and apex resection (AR). (**A**) Instruments and utensils required for AR and sham surgery in zebrafish, details are specified in materials and methods. (**B**–**J**) Pictures demonstrating each major step in AR surgery (See Supplementary Video S2 for AR and Supplementary Video S3 for sham surgery). (**B**) Locating the beating heart. (**C**) Use the forceps to gently lift up the skin. (**D**) With the micro scissors make a small incision just above the heart. (**E**) Widen the incision with the forceps. (**F**) Gently applied pressure on the sides of the incision will make the heart “pop out”. (**G**) 10–20% of the apex is resected, white arrow marks the resected piece. (**H**) Remove the instruments and let the heart get back in place before wiping away the blood. (**I**) The wound after the procedure. (**J**) The recovering zebrafish (See Supplementary Video S4 for details regarding recovery). (**K**) Haematoxylin and eosin (HE) stained section from sham and AR hearts 2 h post injury (hpi) showing the heart and where the bulbus should be located as well as the fibrin/blood clot at the resection site. Scalebar: K = 300 µm.

**Figure 3:**
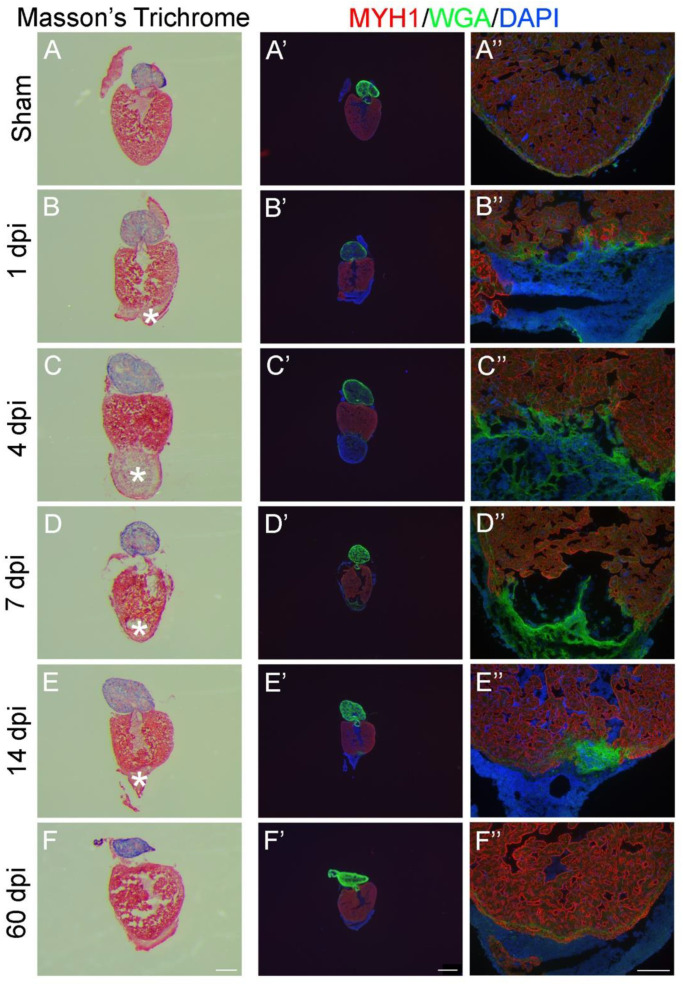
Evaluation of zebrafish heart regeneration in 2D-analysis using histology and immunofluorescence. Cryosections from either sham operated (**A**,**A’**,**A’’**) or apex resected (**B**–**F**, **B’–F’**, **B’**’–**F’’**). Zebrafish hearts were stained with Masson’s Trichrome (**A**–**F**) or immunostained with anti-MYH1 against myosin heavy chain (MYH1, red), wheat germ agglutinin (WGA, green) and 4′,6′-diamidino-2-phenylindole (DAPI, blue) (**A’**–**F’** and **A’’**–**F’’**). Five timepoints (dpi: days post injury) after apex resection were investigated: 1 dpi (**B**,**B’**,**B’**’), 4 dpi (**C**,**C’**,**C’’**), 7 dpi (**D**,**D’**,**D’’**), 14 dpi (**E**,**E’**,**E’’**) and 60 dpi (**F**,**F’**,**F’’**). * marks fibrin clot. Scalebars: A–F = 300 µm, A’–F’ = 500 µm and A’’–F’’ = 100 µm.

**Figure 4:**
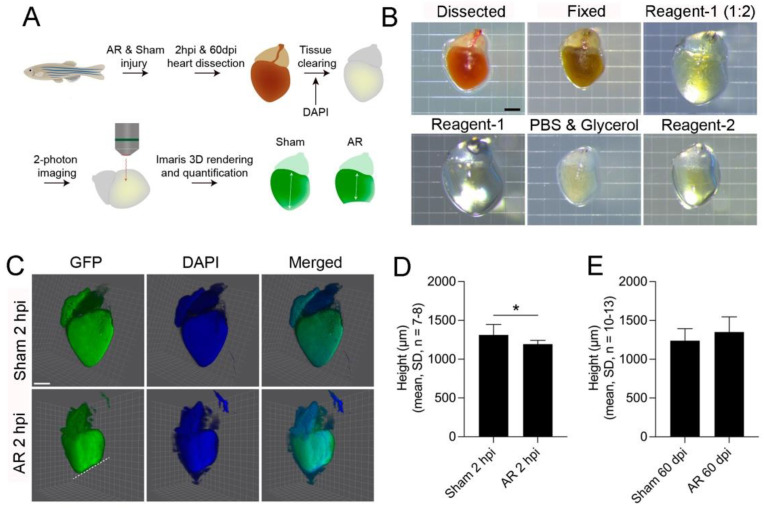
Quantification of heart outgrowth using 3D-analysis of whole zebrafish hearts. (**A**) Schematic of the experiment design from heart dissection through tissue clearing to Imaris analysis of 2-photon images. (**B**) Stereomicroscopy of a zebrafish heart during the different stages of tissue clearing with ScaleCUBIC reagent-1 and -2 [29]. Scale bar: 500 µm. (**C**) 3D images of sham operated or apex resected *tg(myl7:EGFP)* zebrafish hearts, expressing green fluorescense protein (GFP) under the control of the cardiac myosin light chain 2 promoter, thus marking the myocardium (green) 2 h post injury (hpi). Hearts were co-stained with 4′,6′-diamidino-2-phenylindole (DAPI, blue) and images generated by Imaris. Scale bar: 500 µm. (**D**,**E**) Based on the IMARIS analysis, the height of the heart was measured from base to apex in sham operated and apex resected zebrafish hearts, (**D**) 2 hpi and (**E**) 60 days post injury (dpi). Statistical analysis included: Normality was tested followed by a Student’s *t*-test, * *p* ≤ 0.05.

To ease the entry for beginners into the AR procedure we generated detailed videos of anesthetizing the zebrafish (Supplementary Video S1), the AR procedure (Supplementary Video S2), the control sham surgery (Supplementary Video S3) and zebrafish recovery (Supplementary Video S4). The instruments required (Figure 2A) are relatively simple but the microsurgical scissor especially should be of high quality to achieve a sufficient resection. The main steps of the AR procedure are as follows (Figure 2B–J,): (1) Anesthetise the zebrafish (Supplementary Video S1) and place it in the pre-cut slit in the holding sponge using the plastic spoon, and locate the beating heart (Figure 2B); (2) Use forceps to lift up the skin (Figure 2C); (3) Make a small incision with the micro scissors (you may need to clean your instruments from scales) (Figure 2D); (4) Widen the incision using forceps (Figure 2E); (5) Gently press down to let the heart pop out (Figure 2F); (6) Cut 10–20% of the apex and make sure that a piece of the heart has been removed (if in doubt exclude the zebrafish from further analysis) (Figure 2G white arrow); (7) Let the heart slip back inside and gently wipe away blood (Figure 2H); The wound will close by itself (Figure 2I); (8) Transfer the zebrafish to a recovery aquarium (Figure 2J) and gently help rinse away the anaesthesia from the gills with system water using a Pasteur pipette (Supplementary Video S4). By definition, a sham surgery equalizes the placebo effect of surgery. Since the heart is completely exposed in AR with the pericardium being damaged a sham zebrafish is required for interpretating the results. Here, sham samples undergo the process of skin opening and heart exposure until the heart nearly reaches the blade of the scissors (Supplementary Video S3). In order to achieve a reproducible AR, we sometimes dissect AR and sham hearts 2 h after surgery to visualize resection site and size. Thereby the size and appearance of the intact ventricle in the sham heart may be compared with the AR heart to envisage the resection site (Figure 2K, 2 h post injury (hpi)), which should be clearly visible with a defined boundary to the fibrin/blood clot showing approximately 10–20% resection (Figure 2K). When optimal settings have been achieved, heart regeneration may be studied in a spatiotemporal manner.

### 2.3. 2D- and 3D-Analysis for Heart Regeneration

To assess cardiac regeneration, hearts from sham operated and AR zebrafish (1, 4, 7, 14, and 60 days post injury (dpi)) may be dissected, embedded and sliced for further analysis via Masson Trichrome staining (collagen in blue, myocytes in red, Figure 3A–F) or by staining myofibers with an antibody against a cardiomyocyte marker such as myosin heavy chain (MYH1) (Figure 3A’–F’ and A’’–F’’, red) eventually combined with a membrane stain (wheat germ agglutinin (WGA)) (Figure 3A’–F’ and A’’–F’’, green). The progression from the fibrin clot to the regenerated apex at day 60 may then be studied (Figure 3). Of specific interest in the field of heart regeneration, cardiomyocyte proliferation may be evaluated in this set-up by merging it with other staining, e.g., against nuclear markers of cardiomyocytes like Mef2 [6] or the proliferating cell nuclear antigen (PCNA) [6,26]. Alternatively, 5-Ethynyl-2′-deoxyuridine (EdU) may be pulse-chased in the living zebrafish to analyse DNA synthesis in dividing cells [27] and then visualized afterwards by staining.

Alternatively to these 2D-analyses, we have speculated on how to appreciate the 3-dimensional structure when estimating heart outgrowth after AR, and herein performed tissue clearing combined with 2-photon confocal imaging of zebrafish hearts 2 h and 60 days after surgery (Figure 4A). By tissue clearing the opaque heart tissue become transparent through the dehydration and removal of lipids (Figure 4B). As described by others [28,29], this increases the refractive index and reduces light scattering allowing for advantageous imaging of the whole sham and AR hearts (Figure 4C). Whereas the cleared heart may be stained with antibodies or probes, we instead utilized the *tg(myl7:EGFP)* zebrafish, which specifically express GFP under the control of the *cardiac myosin light chain* (*myl7*) 2 promoter in cardiomyocytes of the myocardium, although the remaining tissue was visualized through DAPI staining (Figure 4C). Accordingly, the resection site was clearly identified in the 3D reconstructed hearts, and we then retrieved the height from the base to the apex of the ventricle. Thus, we found the height from base to apex to be shorter in 2 hpi AR hearts as compared to sham hearts (Figure 4D) but did not observe a significant difference at day 60 (Figure 4E). This suggests that the zebrafish hearts have regenerated their myocardium to an extent where no difference to sham hearts may be detected, and opens up the use of advanced imaging for quantifying heart outgrowth after AR.

## 3. Discussion

AR is a potent model and tool for investigating the cardiac regenerative process, however there has been a need for standardization to facilitate interstudy reproducibility. Others have described the technique in detail [6,21], however until now no videos of the procedure have been publicly available. Here we have provided videos of both the sham procedure and AR to ease entry for beginners in the field, but also with the hope of spurring further interest in standardizing the technique. Even though the field of zebrafish heart research seems largely aligned, we encountered interstudy differences in our recent systematic review of AR studies. In particular, the inclusion of shams at each timepoint may be requested to adjust for any (time dependent) effects from the peri-epicardium [10] that is damaged when opening up the thoracic cavity. In many studies, the sham is poorly described, which might lead to confusion regarding what constitutes an appropriate sham control [20]. We have included a video of what we suggest is an optimal sham for AR in zebrafish. Another aspect when further standardizing the AR includes enhancing zebrafish survival, which is especially evident with regards to the sensitivity of zebrafish towards anaesthesia as we show herein. Indeed, we observed marked differences in zebrafish reflex repression, recovery and mortality based on anaesthesia concentration, duration, and bodyweight, and which also depend on the zebrafish strain [22,30] and age [23,31], and thus should be carefully adjusted when setting up the AR zebrafish model.

Whereas the process of heart regeneration in zebrafish hearts often is evaluated in 2D as also performed herein, we hope to inspire others to come up with new ideas on how to analyse heart outgrowth using the more unbiased 3D. Since the histological and immunological approaches all bring valuable information on the regeneration process, the selection of heart tissue sections may bias the analysis. For instance, if one chooses to analyse the height of the ventricle to estimate the myocardial outgrowth, the sections used should embrace the middle and largest part of the heart in a 2D view. Alternatively, our approach with 3D-analysis of tissue cleared hearts may offer a more accurate assessment of where the resection site resides and how it is oriented. This may also enable better quantification and comparison of both height and volumes between AR and sham hearts. Herein we only analysed the height of the myocardium which was defined by the cardiomyocyte GFP expression. Yet the volume may represent an even better parameter. In a pioneering study the Mercader group recently used tissue clearing and subsequent confocal analysis to estimate Sox10+ cardiomyocytes in zebrafish hearts [32]. Nevertheless, the utilization of 3D-based techniques for quantifying heart outgrowth after AR is generally lacking. Yet since the biological variation in zebrafish heart size is relatively high, we believe that the 3D-method presented herein may be improved by normalizing to zebrafish length or weight and it likely also needs to be finetuned with respect to the sex of the animal. We do however consider it valuable to combine both 2D and 3D methods to gain as much knowledge on the heart outgrowth after AR as possible. Besides the regenerative process concerning cardiomyocyte proliferation, the overall regeneration is much more complex and requires consorted actions from various angles that may also be studied using the AR model. Hence vascularization and electrical coupling of the cardiomyocytes [3,4,8], as well as crucial actors like fibroblasts, endothelial cells, smooth muscle cells and the extracellular matrix may also be investigated.

Yet, the AR model is also limited in the sense that it lacks ischemia-induced cell death or cell debris that needs clearing, which is characteristic of MI in humans. Alternatively, heart cryoinjury in zebrafish contains these aspects although the process of heart regeneration requires a longer time before full recovery [33,34]. Other limitations of using zebrafish as a model for mammalian cardiac regeneration includes the fact that fibrin fibers in the zebrafish do not resemble post-MI mammalian scarring, zebrafish only have limited collagen deposition [3,34], and there are obvious differences in cardiac physiology, number of atria and ventricles, and a lack of genuine coronary arteries in the zebrafish.

Despite these differences the AR model is an attractive model for studying heart regeneration, especially to gain insight into mechanistic differences between mammals and zebrafish, which may be translated back into mammals for improving repair after MI. We thus hope the present work will ease the implementation of the AR model for beginners and spur further interest into its standardisation for existing users.

## 4. Materials and Methods

### 4.1. Ethics

All animal experiments were approved by the Danish Animal Inspectorate under the Ministry of Food and Agriculture (J. no.: 2016-15-0201-00874).

### 4.2. Zebrafish

Outbred wild type (AB) or *tg(myl7:EGFP)* [35] zebrafish aged 5–9 months were used. Zebrafish were obtained from the NNF Center for Stem Cell Biology (DanStem), University of Copenhagen and bred either at DanStem or at DCA-lab, Institute of Clinical Research, University of Southern Denmark. Zebrafish were housed in a 14/10 light/dark cycle and fed twice a day.

### 4.3. Materials Required for Apex Resection (AR) in Zebrafish

- 2 small aquariums/tanks for sedation and recovery
- Small fishing net
- Microscope (e.g., Leica, S9i)
- Large plastic spoon
- Pasteur pipette
- Fine curved forceps (S&T, Agnthos, Lidingo, Sweden, cat. no. JFCL-3P TC)
- Micro scissors (LAWTON, Fridingen an der Donau, Germany, cat. no. 05-0050-C)
- Sponge with pre-cut slit big enough to hold the zebrafish
- Pro-ophta, pre-cut on the middle in a 30° angle/cotton swab
- Tissues for removing (zebrafish) scales from surgical instruments
- Tricaine solutions in water from your system/aquarium for anaesthesia
- Water from your system/aquarium

### 4.4. Tricaine Stock Solution

Stock solutions of Tricaine were established by dissolving Ethyl 3-aminobenzoate methanesulfonate (Sigma-Aldrich, Søborg, Denmark, cat. no: E10521) in ddH_2_O, the pH was thereafter adjusted to 7.4 with 1M Tris-base (Sigma-Aldrich, cat. no: T1503) in ddH_2_O. Stock solution was kept at −20 °C for a shorter time until use.

### 4.5. Anaesthesia of Zebrafish

The zebrafish was placed in either 0.02%, 0.03%, or 0.04% Tricaine in system water for either 120, 150, or 180 s to reach stage 3 anaesthesia for the initial part of the study. For the remaining part of the study 0.03% Tricaine for 120 s was used. Hereafter the zebrafish was tested for loss of tail reflex before it was placed in the pre-cut slit in the sponge (Supplementary Video S1).

### 4.6. Surgical Procedures and Recovery

The sedated zebrafish, in the pre-cut slit in the holding sponge, were placed underneath a stereomicroscope (Leica, S9i) with the head of the zebrafish pointing towards the non-dominant hand of the person doing the surgery. Afterwards the sham or AR procedure was performed as shown in Supplementary Videos S3 and S2, respectively. Finally, the zebrafish was placed in the recovery aquarium and monitored during its recovery, while the Tricaine was gently rinsed out of the gills with a Pasteur pipette until gill activity was increased (Supplementary Video S4). After full recovery from anaesthesia the zebrafish was placed back in the system. The general health status of the zebrafish was monitored closely for the first 48 h.

### 4.7. Tissue Processing for 2D Analysis

At the endpoint zebrafish were euthanized by placing them in ice-cold water for 3 min whereafter the hearts were removed, embedded in Tissue-Tek (Sakura, Alphen aan den Rijn, The Netherlands, cat. no.: 4583,) before they were snap-frozen in dry ice-cooled isopentane. Hearts were sectioned in 10 µm thick sections and kept at –80 °C before staining.

### 4.8. Haematoxylin and Eosin Staining

In brief, sections were fixed in 4% neutral buffered formalin (NBF), rinsed in running tap water, stained in Mayer haematoxylin (VWR, Søborg, Denmark, cat. no.: AMPQ12731), rinsed in running tap water and stained in eosin (Sigma-Aldrich, Søborg, Denmark, cat. no.: 1.09844), before they were dehydrated in increasing concentrations of ethanol and mounted with Pertex (Histolab, Askim, Sweden, cat. no.: 00811).

### 4.9. Masson’s Trichrome Staining

Masson’s Trichrome staining was performed as recommended by the manufacturer. Briefly the sections were acclimatized, fixed in Bouin’s solution (Sigma Aldrich, cat. no.: HT10132), incubated with Weigerts Iron Haematoxylin (Sigma-Aldrich, cat. no.: HT109), and then incubated in the Masson’s Trichrome Staining Kit (Sigma-Aldrich, cat. no.: HT15).

### 4.10. Fluorescence staining with Myosin Heavy Chain Antibody (MYH1), Wheat Germ Agglutinin (WGA) and 4′,6′-diamidino-2-phenylindole (DAPI)

Sections were fixed at 10 min in 4% NBF (Sigma-Aldrich cat. no.: HT501128), rinsed in tris-buffered saline (TBS), permeabilized in TBS containing 0.5% Triton-X 100 (Sigma-Aldrich, cat. no.: T8787), rinsed in TBS, blocked for non-specific binding in TBS containing 2% BSA (VWR, cat. no.: 0332) and incubated for 2 h at room temperature with anti-MYH1 (DSHB, Iowa city, IA, USA, Mouse IgG2b,κ, 1:200) and 488-conjugated WGA (Invitrogen, Roskilde, Denmark, cat. no.: W11261, 1:100). Following incubation, sections were rinsed in TBS and incubated with a secondary antibody (Invitrogen, cat. no.: A31570, 1:200) before a final rinse in TBS and mounted with antifade mounting medium with DAPI (Vectarshield, Vector Laboratories, Burlingame, CA, USA, cat. no.: H-1200).

### 4.11. CUBIC Tissue Clearing and 2-Photon Imaging

Zebrafish were euthanized as above and hearts were carefully dissected, whereafter they were placed in 2–3 drops of phosphate buffered saline (PBS). The atrium was then removed, before the hearts were placed in individual wells in a 24-well plate containing 4% NBF for 24 h at 4 °C. On the following day the hearts were incubated in 1:2 dilution of ddH_2_O and ScaleCUBIC-1 reagent [29] for 15 min, whereafter the hearts were transferred to pure ScaleCUBIC-1 reagent with DAPI (1:1000) and incubated overnight, shaking at approximately 80 rpm at 37 °C. If the ventricle didn’t appear transparent/yellowish after overnight incubation the incubation period was prolonged (if prolonged for more than 24 h, fresh ScaleCUBIC-1 reagent was added). Following incubation in ScaleCUBIC-1 reagent the hearts were washed 3 × 1.5 h in 1× PBS, shaking at approximately 80 rpm at room temperature, before they were incubated overnight, shaking at approximately 80 rpm at room temperature in a 1:2 dilution of glycerol (Merck, cat. no: 8.18709.1000) and PBS. Finally, the hearts were incubated, shaking at approximately 80 rpm at 37 °C overnight in ScaleCUBIC-2 reagent [29]. If the clearing process was incomplete the incubation period was prolonged (if prolonged for more than 24 h, fresh ScaleCUBIC-2 reagent was added). After tissue clearing, multiphoton imaging was performed using an Olympus FV1000 MPE multiphoton laser scanning microscope, fitted with a Titanium Saphire) Mai-Tai Deep See laser (690–1040 nm) and an Olympus UPLXAPO 4X/0.16NA objective. Images were acquired with standardized image settings with a resolution of 2048 × 2048 pixels and a scan speed of 4.0 us/pixel, and software optimized numbers of Z-stacks was determined for each heart (~30–60 stacks). DAPI excitation was performed at 800 nm and captured within the emission spectra of 420–520 nm. GFP excitation was performed at 895 nm and captured within the emission spectra of 515–560 nm. Image analysis and 3D image reconstruction of the obtained Tiff image stacks were performed using the Imaris software (Bitplane Scientific Software; version 9.6) in blend mode. Myocardial height was measured from the apex to base of the ventricle as defined by GFP expression.

### 4.12. Statistical Analysis

Comparisons were performed using one- and two-way ANOVA with Tukey’s multiple post-test (Figure 1) or using the Student’s *t*-test (Figure 4) (GraphPad Software, San Diego, CA, USA). Statistical significance was established for *p* ≤ 0.05. Data are presented as mean ± SD.

## Data Availability

The data that support the findings of this study are available from the corresponding author upon reasonable request.

## Supplementary Materials

The following are available online at https://www.mdpi.com/article/10.3390/ijms22115865/s1, Video S1: Anesthesia; Video S2: AR; Video S3: Sham; Video S4: Recovery.

## References

[1] van Amerongen M.J., Engel F.B. (2008). Features of Cardiomyocyte Proliferation and its Potential for Cardiac Regeneration. J. Cell. Mol. Med..

[2] Burke A.P., Virmani R. (2007). Pathophysiology of Acute Myocardial Infarction. Med. Clin. N. Am..

[3] Poss K.D., Wilson L.G., Keating M.T. (2002). Heart Regeneration in Zebrafish. Science.

[4] Raya A., Koth C.M., Buscher D., Kawakami Y., Itoh T., Raya R.M., Sternik G., Tsai H.-J., Rodríguez-Esteban C., Izpisúa-Belmonte J.C. (2003). Activation of Notch Signaling Pathway Precedes Heart Regeneration in Zebrafish. Proc. Natl. Acad. Sci. USA.

[5] González-Rosa J.M., Mercader N. (2012). Cryoinjury as a Myocardial Infarction Model for the Study of Cardiac Regeneration in the Zebrafish. Nat. Protoc..

[6] Wang J., Poss K.D. (2016). Methodologies for Inducing Cardiac Injury and Assaying Regeneration in Adult Zebrafish. Methods Mol. Biol..

[7] Howe K., Clark M.D., Torroja C.F., Torrance J., Berthelot C., Muffato M., Collins J.E., Humphray S., McLaren K., Matthews L. (2013). The Zebrafish Reference Genome Sequence and its Relationship to the Human Genome. Nature.

[8] Lepilina A., Coon A.N., Kikuchi K., Holdway J.E., Roberts R.W., Burns C.G., Poss K.D. (2006). A Dynamic Epicardial Injury Response Supports Progenitor Cell Activity during Zebrafish Heart Regeneration. Cell.

[9] Kikuchi K., Holdway J.E., Major R.J., Blum N., Dahn R.D., Begemann G., Poss K.D. (2011). Retinoic Acid Production by Endocardium and Epicardium is an Injury Response Essential for Zebrafish Heart Regeneration. Dev. Cell.

[10] Wang J., Cao J., Dickson A.L., Poss K.D. (2015). Epicardial Regeneration is Guided by Cardiac Outflow Tract and Hedgehog Signalling. Nat. Cell Biol..

[11] Jopling C., Sleep E., Raya M., Marti M., Raya A., Izpisua Belmonte J.C. (2010). Zebrafish Heart Regeneration Occurs by Cardi-omyocyte Dedifferentiation and Proliferation. Nature.

[12] Kikuchi K., Holdway J.E., Werdich A.A., Anderson R.M., Fang Y., Egnaczyk G.F., Evans T., MacRae C.A., Stainier D., Poss K.D. (2010). Primary Contribution to Zebrafish Heart Regeneration by gata4+ Cardiomyocytes. Nat. Cell Biol..

[13] Kikuchi K., Gupta V., Wang J., Holdway J.E., Wills A.A., Fang Y., Poss K.D. (2011). tcf21+ Epicardial Cells Adopt Non-Myocardial Fates during Zebrafish Heart Development and Regeneration. Development.

[14] Senyo S.E., Steinhauser M.L., Pizzimenti C.L., Yang V.K., Cai L., Wang M., Wu T.-D., Guerquin-Kern J.-L., Lechene C.P., Lee R.T. (2013). Mammalian Heart Renewal by Pre-Existing Cardiomyocytes. Nat. Cell Biol..

[15] Bersell K., Arab S., Haring B., Kühn B. (2009). Neuregulin1/ErbB4 Signaling Induces Cardiomyocyte Proliferation and Repair of Heart Injury. Cell.

[16] Bergmann O., Bhardwaj R.D., Bernard S., Zdunek S., Barnabé-Heider F., Walsh S., Zupicich J., Alkass K., Buchholz B.A., Druid H. (2009). Evidence for Cardiomyocyte Renewal in Humans. Science.

[17] Bargehr J., Ong L.P., Colzani M., Davaapil H., Hofsteen P., Bhandari S., Gambardella L., Le Novère N., Iyer D., Sampaziotis F. (2019). Epicardial Cells Derived from Human Embryonic Stem Cells Augment Cardiomyocyte-Driven Heart Regeneration. Nat. Biotechnol..

[18] Huang G., Thatcher J.E., McAnally J., Kong Y., Qi X., Tan W., DiMaio J.M., Amatruda J.F., Gerard R.D., Hill J.A. (2012). C/EBP Transcription Factors Mediate Epicardial Activation During Heart Development and Injury. Science.

[19] Smart N., Bollini S., Dubé K.N., Vieira J.M., Zhou B., Davidson S., Yellon D., Riegler J., Price A.N., Lythgoe M. (2011). De novo Cardiomyocytes from within the Activated Adult Heart after Injury. Nat. Cell Biol..

[20] Belling H.J., Hofmeister W., Andersen D.C. (2020). A Systematic Exposition of Methods used for Quantification of Heart Regeneration after Apex Resection in Zebrafish. Cells.

[21] Sheng D.Z., Zheng D., Kikuchi K. (2020). Cardiac Resection Injury in Zebrafish. Methods in Molecular Biology.

[22] Collymore C., Tolwani A., Lieggi C., Rasmussen S. (2014). Efficacy and Safety of 5 Anesthetics in Adult Zebrafish (*Danio rerio*). J. Am. Assoc. Lab. Anim. Sci..

[23] Carter K.M., Woodley C.M., Brown R.S. (2011). A Review of Tricaine Methanesulfonate for Anesthesia of Fish. Rev. Fish Biol. Fish..

[24] Valentim A.M., Félix L.M., Carvalho L., Diniz E., Antunes L.M. (2016). A New Anaesthetic Protocol for Adult Zebrafish (*Danio rerio*): Propofol Combined with Lidocaine. PLoS ONE.

[25] Ehrlich O., Karamalakis A., Krylov A.J., Dudczig S., Hassell K.L., Jusuf P.R. (2019). Clove Oil and AQUI-S Efficacy for Zebrafish Embryo, Larva, and Adult Anesthesia. Zebrafish.

[26] Narumanchi S., Kalervo K., Perttunen S., Wang H., Immonen K., Kosonen R., Laine M., Ruskoaho H., Tikkanen I., Lakkisto P. (2019). Inhibition of let-7c Regulates Cardiac Regeneration after Cryoinjury in Adult Zebrafish. J. Cardiovasc. Dev. Dis..

[27] Salic A., Mitchison T.J. (2008). A Chemical Method for Fast and Sensitive Detection of DNA Synthesis In vivo. Proc. Natl. Acad. Sci. USA.

[28] Ariel P. (2017). A Beginner’s Guide to Tissue Clearing. Int. J. Biochem. Cell Biol..

[29] Susaki A.E., Tainaka K., Perrin D., Yukinaga H., Kuno A., Ueda H. (2015). Advanced CUBIC Protocols for Whole-Brain and Whole-Body Clearing and Imaging. Nat. Protoc..

[30] Martins T., Diniz E., Félix L.M., Antunes L. (2018). Evaluation of Anaesthetic Protocols for Laboratory Adult Zebrafish (*Danio rerio*). PLoS ONE.

[31] Matthews M., Varga Z.M. (2012). Anesthesia and Euthanasia in Zebrafish. ILAR J..

[32] Sande-Melón M., Marques I.J., Galardi-Castilla M., Langa X., Pérez-López M., Botos M.A., Sánchez-Iranzo H., Guz-mán-Martínez G., Ferreira Francisco D.M., Pavlinic D. (2019). Adult sox10(+) Cardio-Myocytes Contribute to Myocardial Regeneration in the Zebrafish. Cell Rep..

[33] González-Rosa J.M., Burns C.E. (2017). Zebrafish Heart Regeneration: 15 Years of Discoveries. Regeneration.

[34] González-Rosa J.M., Martín V., Peralta M., Torres M., Mercader N. (2011). Extensive Scar Formation and Regression during Heart Regeneration after Cryoinjury in Zebrafish. Development.

[35] Huang C.-J., Tu C.-T., Hsiao C.-D., Hsieh F.-J., Tsai H.-J. (2003). Germ-Line Transmission of a Myocardium-Specific GFP Transgene Reveals Critical Regulatory Elements in the Cardiac Myosin Light Chain 2 Promoter of Zebrafish. Dev. Dyn..

